# Supplementary material for: Reporter Assays for Ebola Virus Nucleoprotein Oligomerization, Virion-Like Particle Budding, and Minigenome Activity Reveal the Importance of Nucleoprotein Amino Acid Position 111
Source: Viruses. 2020 Jan 15;12(1):105. doi: 10.3390/v12010105 (PMC7019320; doi:10.3390/v12010105)
Supplement: Supplementary file 1 [file viruses-12-00105-s001.zip › supplementary/Supplementary-Figures.pdf]

viruses

MDPI

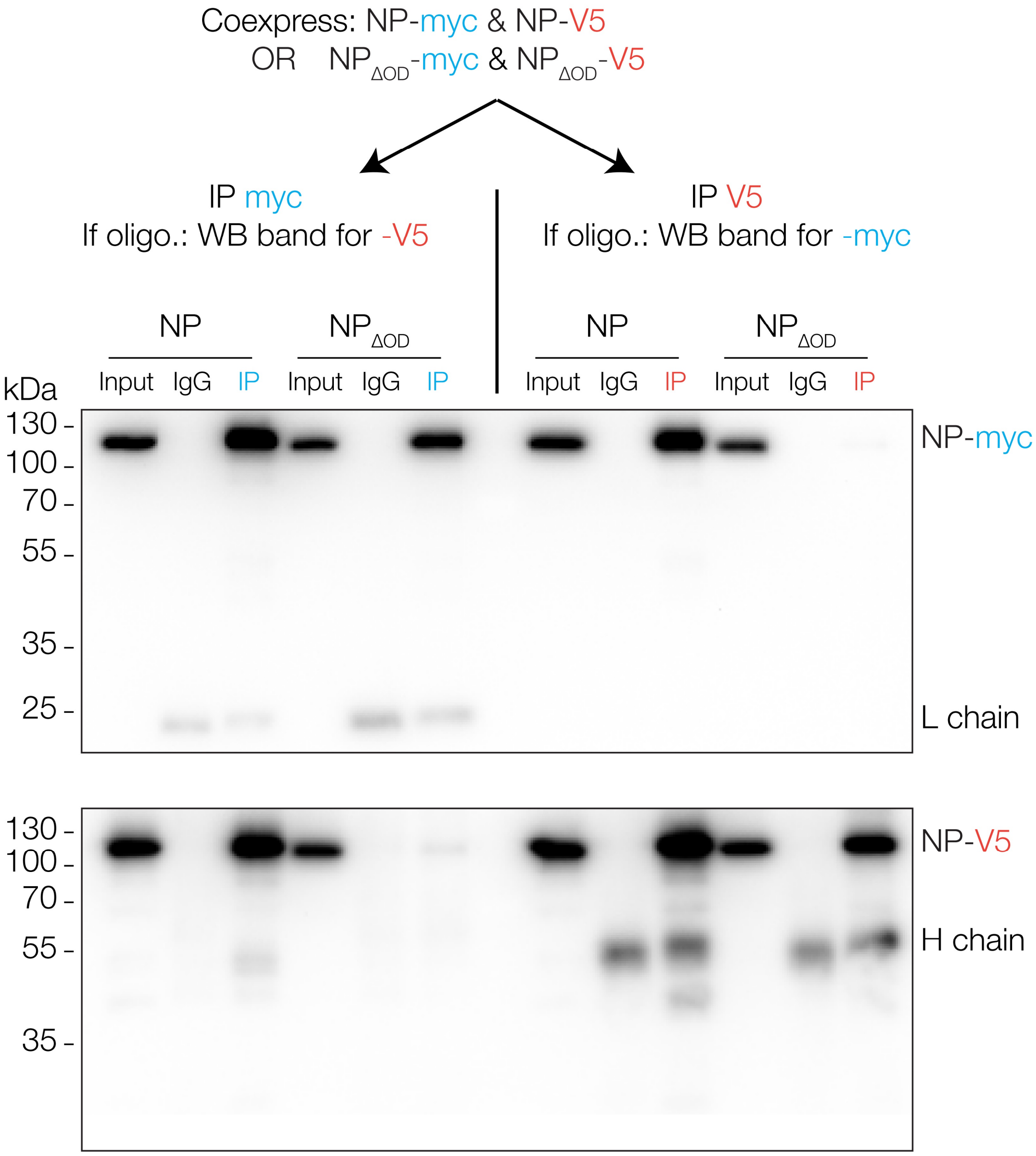

Figure S1. Deletion of the reported NP oligomerization domain (NP-ΔOD) prevents NP oligomerization in cell culture. We co-expressed myc- and V5-tagged NP or NP-ΔOD (deletion of residues 20–38) in HEK 293FT cells, harvested and lysed cells, and performed co-immunoprecipitation (co-IP) targeting either the myc (blue) or the V5 (orange) tag, or IgG isotype controls. We used 2.5% of each input and 25% of each IP for detection of co-eluting proteins by WB for each tag. Oligomerization is indicated by heterologous detection (NP-V5 band in the IP myc fraction, and vice versa) with full-length NP, which did not occur with NP-ΔOD. Additional bands are due to heavy (H) and light (L) chains of the co-IP antibody.

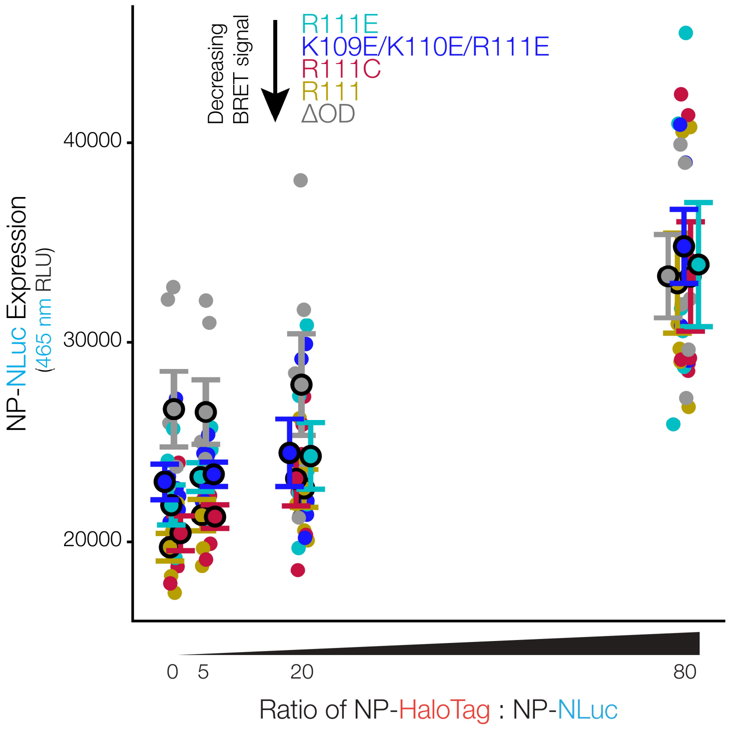

Figure S2. NP-NLuc variants express at similar concentrations. As part of the BRET assay, we measured NLuc-driven luminescence of each NP genotype in the absence of HaloTag ligand in HEK 293FT cells. This luminescence solely reflects the amount of NP-NLuc expression since no resonant energy transfer can occur without the HaloTag ligand. Coloring is the same as in Figure 3D. NP-NLuc expression does not appear to correlate with oligomerization propensity. Error bars indicate mean ± standard error of the mean (SEM).

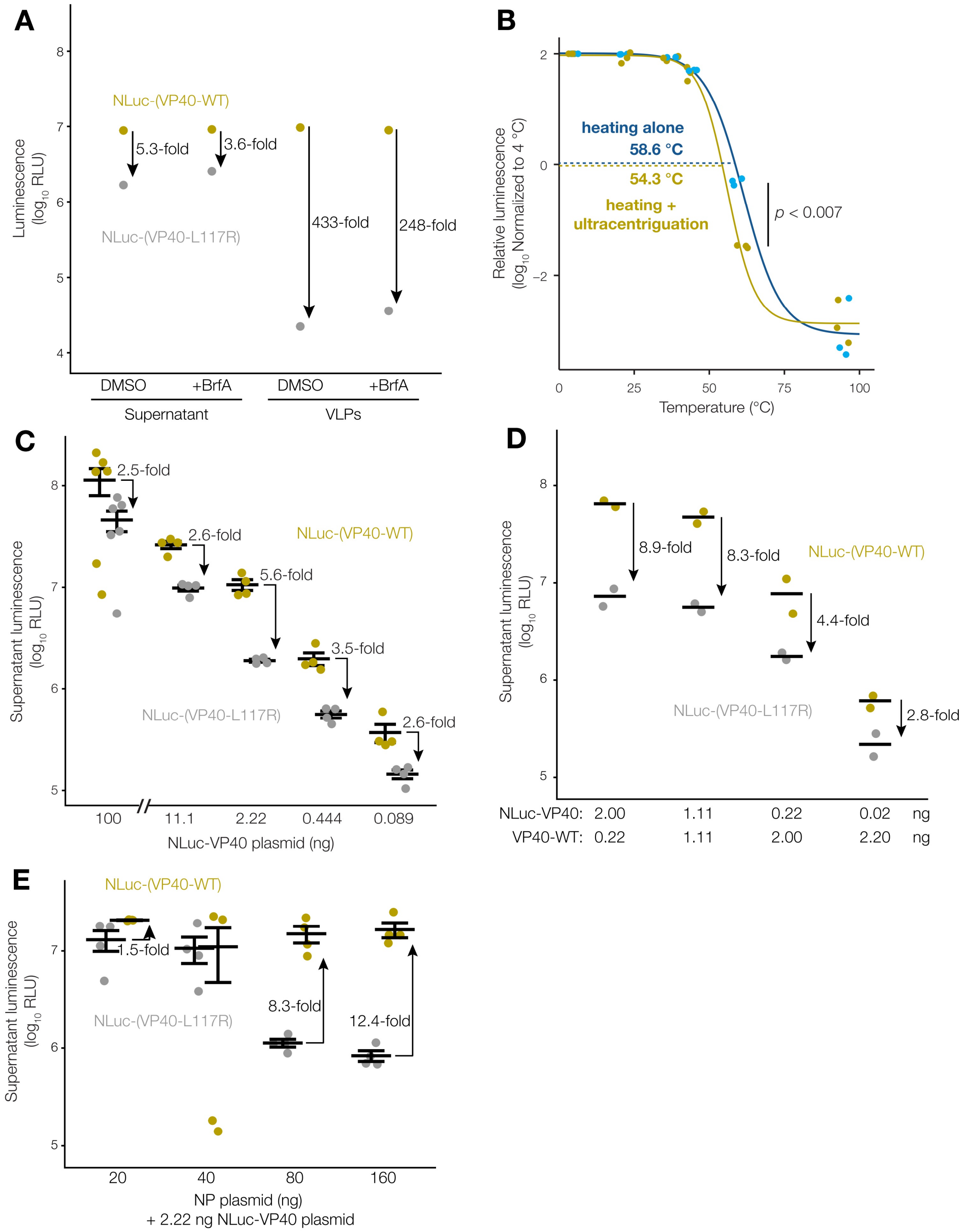

Figure S3. Optimization of NLuc-VP40 VLP assay. (**a**) VP40 monomers are expelled from cells independent of coat protein complex I (COPI) transport. We expressed NLuc-VP40 wild-type (tan) or L117R substitution (gray) in HEK 293FT cells, and treated with either brefeldin A (BrfA) or dimethyl sulfoxide (DMSO) vehicle control. We collected culture supernatant and measured luminescence directly, or purified VLPs through a 20% sucrose cushion and then measured luminescence. NLuc-(VP40-L117R) luminescence greatly decreased after ultracentrifugation, suggesting that VP40 in culture supernatant was monomeric. BrfA did not appear to affect Nluc-(VP40-L117R) luminescence, suggesting that this phenomenon is independent of COPI transport; (**b**) Heating supernatant prior to ultracentrifugation results in loss of VLP luminescence. We expressed Nluc-VP40 in cells, collected total supernatant, and heated at various temperatures. We then either measured luminescence immediately to assess Nluc thermal stability (blue), or pelleted VLPs and then measured luminescence to assess VLP stability (tan) (n = 3 biological replicates). We normalized luminescence at each temperature to the 4 °C condition, log-transformed, and fitted to sigmoidal

curves (Equation 3), and calculated statistical significance by paired *t*-test; (**c**) Lower amounts of NLuc-VP40 plasmid led to more VLP budding than monomer expulsion. We expressed NLuc-VP40 wild-type (tan) or L117R mutant (gray) in cells, collected culture supernatant, and measured NLuc luminescence (n = 4–6 biological replicates). We observed the largest difference between NLuc-VP40 and NLuc-(VP40-L117R) at 2.22 ng plasmid per well of a 96-well plate. Error bars indicate mean ± SEM; (**d**) Co-expressing untagged VP40 in cells does not improve NLuc-VP40 VLP budding. We co-expressed NLuc-VP40 wild-type (tan) or L117R mutant (gray) with 'dark' untagged VP40 in cells, and measured culture supernatant luminescence (n = 2 biological replicates). Increasing amounts of 'dark' VP40 did not increase the difference between the NLuc-VP40 and NLuc-(VP40-L117R). Error bars indicate mean; (**e**) Expression of NP increases VLP production. We co-expressed NLuc-VP40 wild-type (tan) or L117R (gray) with NP in cells, collected culture supernatant, and measured culture supernatant luminescence (n = 4 biological replicates). Increasing amounts of NP plasmid increased the difference between VP40 variants. Error bars indicate mean ± SEM.

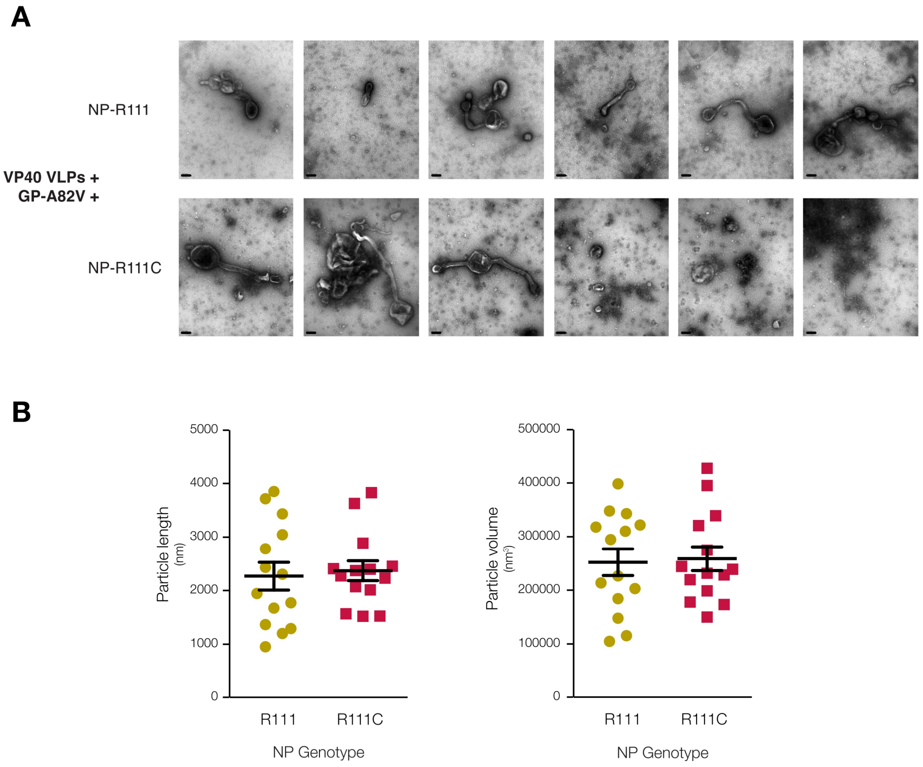

Figure S4. Electron microscopy of NP-R111 and NP-R111C VLPs. (**a**) EM of VP40 VLPs created by with co-expression of VP40, GP-A82V, and NP-R111 or NP-R111C in HEK 293T cells. We elected to use GP with the A82V substitution since the NP-R111C substitution occurred on the GP-A82V background (Figure 1A). Scale bar (black line in bottom left) denotes 200 nm. Error bars indicate mean ± SEM; (**b**) Quantification of VLP size and volume for VLPs. VLPs bearing NP-R111 (tan) and NP-R111C (red) have similar size and volume. Error bars indicate mean ± SEM.

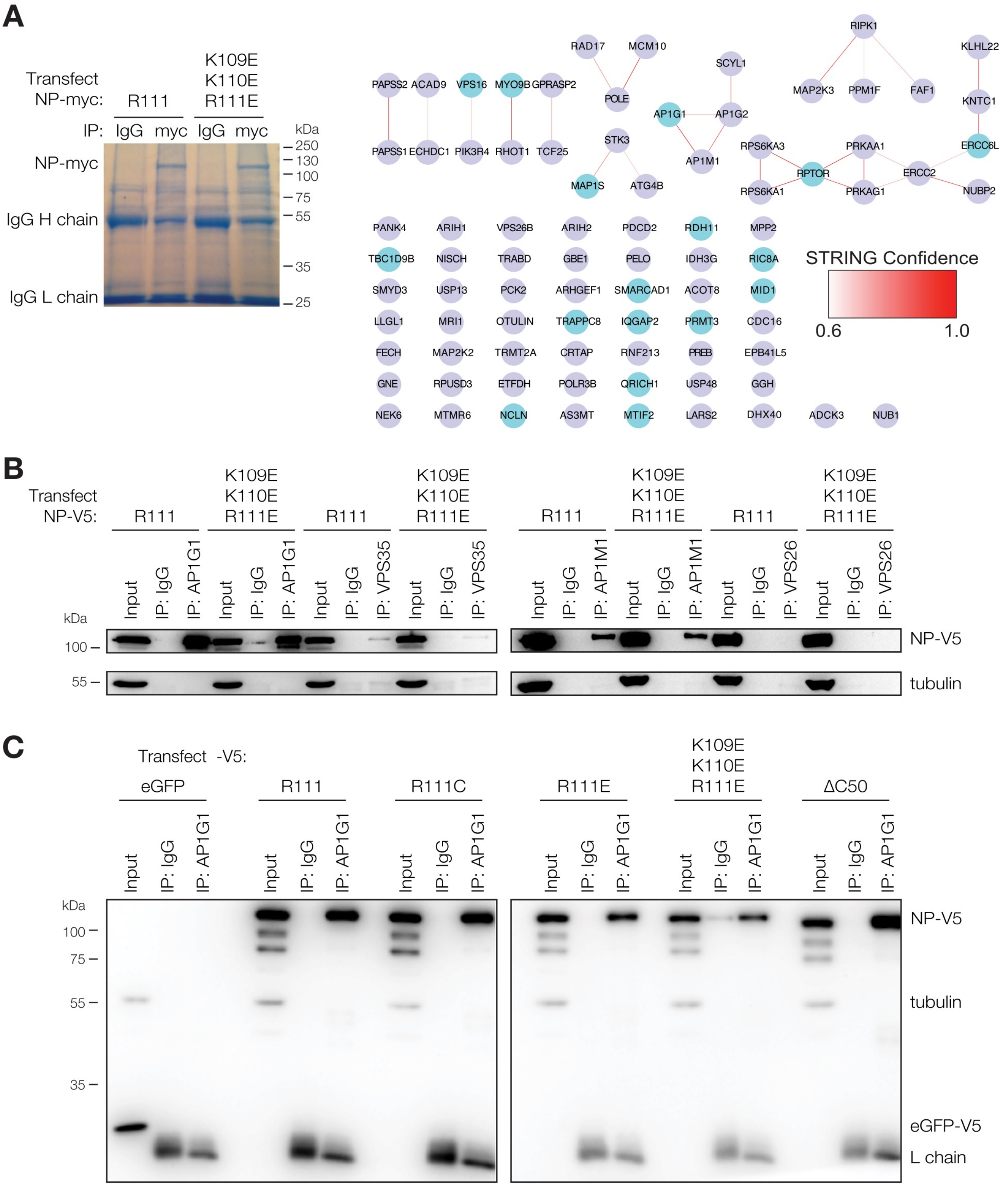

Figure S5. EBOV NP interacts with the adaptor protein 1 (AP-1) complex independent of the NP 111 allele. (**a**) IP-MS/MS and STRING analysis of proteins interacting with NP-myc harboring ancestral R111 or triple charge-reversal (K109E/K110E/R111E) in HEK 293FT cells. Light blue nodes: proteins enriched in both replicates. Darker red lines: high confidence STRING interactions; lighter red: low confidence. See also File S2; (**b**) Reciprocal co-IP of NP with AP-1 and vacuolar protein sorting (VPS) antibodies. Because there was more total protein in the input than the IP fraction, we loaded 5% of each input and 50% of each IP. Adaptor related protein complex 1 subunit gamma 1 (AP1G1), and mu 1 (AP1M1) are strong interactors of NP, whereas VPS35 is one of several weak interactors; (**c**) Reciprocal co-IP of all NP mutants and eGFP negative control with α-AP1G1 antibody. We loaded 5% of each input and 50% of each IP. No apparent differences were observed between any of the NP mutants.
